# Stress hyperglycemia increases short-term mortality in acute ischemic stroke patients after mechanical thrombectomy

**DOI:** 10.1186/s13098-024-01272-5

**Published:** 2024-01-31

**Authors:** Bing Yang, Xuefang Chen, Fangze Li, Junrun Zhang, Dawei Dong, Huiyue Ou, Longyan Lu, Niu He, Xiaohong Xu, Xiufeng Xin, Jingchong Lu, Min Guan, Hongyu Qiao, Anding Xu, Huili Zhu

**Affiliations:** 1 Department of Neurology and Stroke Center, The First Affiliated Hospital, Jinan University, 613 Huangpu Avenue west, Guangzhou, China; 2 Clinical Neuroscience Institute, Jinan University, Guangzhou, China; 3 Department of Neurology, The Dongguan Affiliated Hospital of Jinan University, Binhaiwan Central Hospital of Dongguan, Dongguan, China; 4 Department of Neurology, the Affiliated Shunde Hospital of Jinan University, Foshan, China

**Keywords:** Acute ischemic stroke, Stress hyperglycemia ratio, Mortality, Mechanical thrombectomy

## Abstract

**Background and purpose:**

Glucose-to-glycated hemoglobin ratio (GAR) is considered a more reliable marker of stress hyperglycemia by correcting for basal blood glucose levels. This study aimed to investigate the extent to which GAR is associated with 3 month and 1 year all-cause mortalities in patients with acute ischemic stroke (AIS) undergoing mechanical thrombectomy (MT).

**Methods:**

We retrospectively followed 553 AIS patients who underwent MT. The degree of stress hyperglycemia was quantified as the GAR, defined as fasting plasma glucose (mmol/L)/hemoglobin A1c (HbA1c) (%) on the second day after admission. According to the GAR quartiles, the patients were further categorized into four groups (group 1-group 4). We assessed the association between GAR and all-cause mortalities, clinical outcomes during hospitalization and function outcomes at 3 months. The associations between stress hyperglycemia and all-cause mortalities were analyzed using a Cox proportional-hazards model, while other outcomes were analyzed using multiple logistic regression analysis.

**Results:**

The follow-up lasted a median of 18 months (range 0–66 months). The 3 month mortality rate was 9.58% (n = 53) and the 1 year mortality rate was 18.62% (n = 103). The Kaplan–Meier analysis revealed a significant inverse relationship between GAR and mortality (*P* < 0.001). In the Cox proportional-hazards model at 3 months, compared with group1, group 4 of GAR was associated with a significant increase in the risk of 3 month mortality (hazard ratio [HR] = 4.11, 95% confidence interval [CI] 1.41–12.0, *P* = 0.01) after adjusting for potential covariates. On multivariate logistic regression analysis, GAR was strongly associated with an increased risk of 3 month poor function outcome.

**Conclusions:**

Stress hyperglycemia, quantified by a higher GAR, is associated with all-cause mortality and poor functional outcomes in patients with AIS who undergo MT. Furthermore, GAR may contribute to improving the predictive efficiency of all-cause mortality in patients with AIS after MT, especially short-term all-cause mortality.

## Introduction

Despite the risk of stroke death reducing significantly over the past decade, acute ischemic stroke (AIS) remains the second most common cause of death worldwide, and a leading cause of long-term disability [1]. Recent randomized controlled clinical trials have provided conclusive evidence that mechanical thrombectomy (MT) improves functional outcomes in AIS patients, which is particularly critical for survival [2–4]. MT is recommended in clinical practice guidelines as the treatment of choice for large-vessel occlusion in AIS [5]. AIS can cause neuroendocrine disorders and increase the levels of corticosteroids, catecholamine, glucagon, and blood glucose [6]. Previous studies have found that stress hyperglycemia affects the clinical prognosis of AIS and may increase the risk of death after stroke [7, 8].

There is no exact definition of stress hyperglycemia. Most previous studies have used admission randomized glucose or fasting plasma glucose (FPG) to identify stress hyperglycemia. However, without consideration of the background glucose concentration, the absolute glucose concentration does not accurately reflect the stress condition of acute stroke. The glucose-to-glycated hemoglobin ratio (GAR) may be a better measure of the degree of stress hyperglycemia [9]. GAR quantifies the extent of acute increase in blood glucose based on the baseline blood glucose levels, which can reflect the different background blood glucose situation comparison. Referring to the literature of Merlino G et al., the patients were further categorized into four even groups according to the quartile of GAR [10–12]. A few studies have investigated the association of GAR with the prognosis after MT in AIS patients [10, 13, 14]. So far, no studies have simultaneously investigated and compared the association of GAR and admission fasting glucose with short-and long-term mortality after MT in patients with AIS. This study aimed to explore the predictive role of blood glucose background concentration-based GAR on 3 month mortality and long-term mortality (mainly 1 year mortality) after surgery in AIS patients treated with MT.

## Methods

### Study participants

This retrospective cohort single-center study enrolled consecutive AIS patients with large vessel occlusion, including anterior circulation and posterior circulation, who underwent MT at the First Affiliated Hospital of Jinan University between January 2016 and December 2020. The inclusion criteria for screening in this study include: (i) age ≥ 18 years, (ii) Patients diagnosed as AIS within 24 h of onset, and (iii) Receiving emergency MT. The following exclusion criteria were applied: (i) unsuccessful vascular reperfusion or vital organ failure prior to onset, while successful vascular reperfusion was defined as a modified thrombolysis in cerebral infarction (mTICI) score 2b or 3 [15]at the end of MT. (ii) without data on FPG, hemoglobin A1c (HbA1c) or head computed tomography (CT)/magnetic resonance imaging (MRI) scans, or (iii) lost to follow-up. The study patients were classified into either four groups according to quartiles of GAR (1st quartile < 0.93, 0.93 ≤ 2nd quartile < 1.13, 1.13 ≤ 3rd quartile < 1.39, 1.39 ≤ 4th quartile). The study was approved by the Ethics Committee of the First Affiliated Hospital of Jinan University (protocol number: KYk-2022-007). Due to the retrospective nature of this study, the ethical committee did not require informed consent.

### Data collection

The following data were collected: age, sex, medical history (atrial fibrillation, previous stroke, cardiovascular disease, diabetes, hypertension, and smoking status), intravenous thrombolysis (IVT) before MT, stroke severity, pre-stroke modified Rankin Scale (mRS) score [16], stroke subtypes based on Trial of ORG 10172 in Acute Stroke Treatment (TOAST) classification [17], baseline Alberta Stroke Program Early CT Score (ASPECTS) [18, 19] of anterior circulation strokes or posterior circulation strokes, systolic blood pressure, diastolic blood pressure, onset-to-recanalization time (ORT), and findings of laboratory tests. Stroke severity was determined using the National Institutes of Health Stroke Scale (NIHSS) score at admission. ORT was defined as the duration from the onset to achieving successful recanalization.

### Assessment of stress hyperglycemia

Fasting venous blood samples were drawn within 24 h after admission and an overnight fast (lasting at least 8 h) to measure FPG and HbA1c. FPG was measured using the hexokinase method, and HbA1c was measured using ion-exchange high-pressure liquid chromatography, with GAR quantified as FPG (mmol/L)/HbA1c (%). Patients were divided into four groups based on the groups of GAR. GAR quantifies the degree of blood glucose elevation, with a higher ratio indicating more severe stress hyperglycemia.

### Outcomes and definitions

The primary endpoint was short-term and long-term all-cause mortality. Short-term mortality was defined as death within 3 months after MT. Long-term mortality was defined as death at 1 year after MT. Secondary outcomes included the proportion of patients achieving 3 month poor outcome, early neurological deterioration (END), stroke-associated pneumonia, intracranial hemorrhage (ICH) and symptomatic intracranial hemorrhage (sICH). Three-month poor functional outcomes was defined as a mRS score of 3–6 at 3 months after admission. END was defined as ≥ 4-point increase in the NIHSS score between baseline and 24 h after MT [20, 21]. Stroke-associated pneumonia was collected from medical records, defined as pneumonia that developed in the hospital 48 h after admission, but was neither present nor in the incubation period of infection on admission. ICH was diagnosed as the absence of intracranial hemorrhage on the first brain computed tomography (CT)/magnetic resonance imaging (MRI) scan after the cerebral infarction, but occurred on the second head CT/MRI scan. A second CT/MRI examination was performed to confirm that every patient we included presented with ICH. According to the second European–Australasian Acute Stroke Study (ECASS II) criteria, sICH was diagnosed as ICH associated with neurologic worsening of at least 4 points on the NIHSS [22]. Group 1 was defined as the first stress hyperglycemia ratio quartile. Group 2 was defined as the second stress hyperglycemia ratio quartile. Group 3 was defined as the third stress hyperglycemia ratio quartile. Group 4 was defined as the fourth stress hyperglycemia ratio quartile. In the survival graph, 3 months and 1 year are represented by 90 or 360 days respectively to better show survival status.

### Statistical analysis

Continuous variables were expressed as mean ± standard deviation or median (interquartile range), and comparisons between groups were performed with one-way ANOVA or the Kruskal–Wallis H test as appropriate. Categorical variables were expressed as frequency (percentage), and the χ^2^ test or Fisher’s exact test was used for intergroup comparisons. Group 1 was used as a reference. Kaplan–Meier (KM) survival analysis and Cox proportional hazard regression model were used to analyze the association between GAR and mortality. The Log-rank test was conducted for non-parametric analysis to compare survival distributions between 4 groups. The Cox regression model of 3 month mortality was adjusted for cardiovascular disease (CVD), age, NIHSS score at admission, and hemoglobin. The Cox regression model of 1 year mortality was adjusted for sex, CVD, NIHSS score at admission, age, systolic blood pressure (SBP), hemoglobin and serum creatinine (Scr). Multiple logistic regression analysis was performed to evaluate the association between GAR and END, stroke-associated pneumonia, ICH, sICH and 3 month poor functional outcomes. Baseline variables that were considered clinically relevant or had a univariate relationship with the prognosis were included in a multivariate Cox proportional-hazards model and multivariate logistic regression model. Given the number of available events, the variables included were all carefully selected to ensure the refinement of the final model. The area under the receiver operating characteristic curve (AUC) was used to examine the potential of GAR, FPG, and HbA1c in predicting mortality. All statistical analyses were performed using R software (version 4.1.2) and SPSS (version 27.0). Hazard ratios (HRs) or odd ratios (ORs) and their 95% confidence intervals (CIs) were calculated. A 2-sided *P* < 0.05 was considered to be statistically significant.

## Results

### Study participants and characteristics

**Fig. 1:**
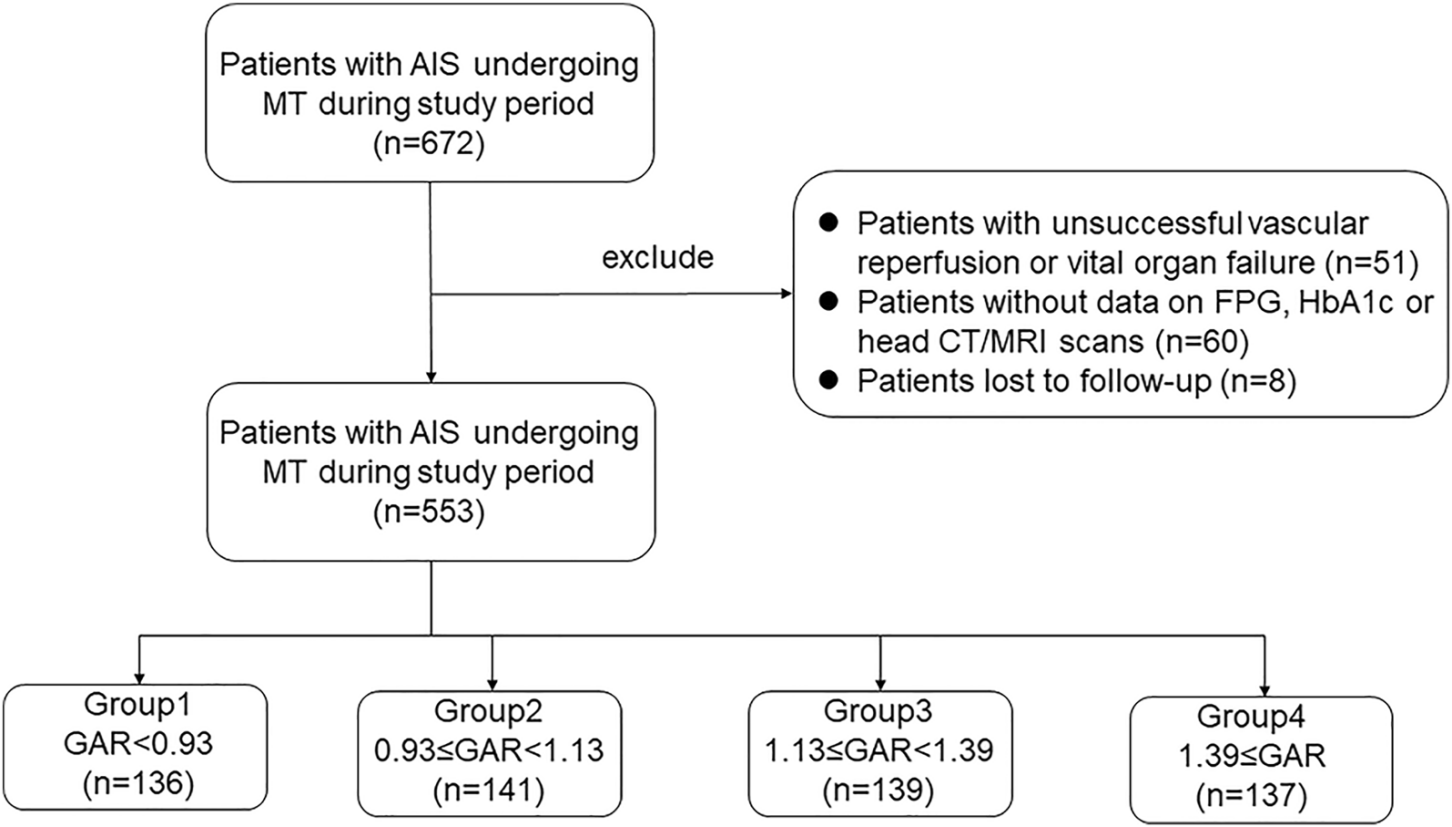
Inclusion and exclusion flowchart of the study. *AIS* acute ischemic stroke, *MT* mechanical thrombectomy, *HbA1c* glycated hemoglobin, *FPG* fasting plasma glucose. Group 1–4 indicate group 1–4 of FPG/HbA1c

During the study period, 672 patients who underwent MT met the inclusion criteria. Of these, 51 patients with unsuccessful vascular reperfusion or vital organ failure, 60 patients without FPG, HbA1c, or head CT/MRI scans, and 8 patients lost to follow-up were excluded. Hence, the study cohort comprised a total of 553 patients with AIS undergoing MT entered the study (Fig. 1). Among these, 435 were anterior circulation strokes and 118 were posterior circulation strokes. According to the quartiles of GAR, they were divided into four groups (group 1-group 4) with 136, 141, 139 and 137 participants, respectively.

The baseline characteristics of study patients (n = 553) are presented in Table 1. In this study, 368 (66.55%) subjects were male and 185 (33.45%) were female. The median age of the included patients was 62 (range 20–94). Patients with higher GAR were significantly older, had higher baseline NIHSS scores, higher admission systolic blood pressures, and higher levels of FPG and HbA1c. History of atrial fibrillation, previous TIA/stroke, diabetes, or hypertension were more common in patients in group 4 of GAR. It was particularly interesting that higher GAR was associated with fewer current smokers. We did not observe any differences in CVD, Pre-stroke mRS, TOAST classification, baseline ASPECTS in anterior circulation strokes or posterior circulation strokes, hemoglobin, high-density lipoprotein (HDL) cholesterol, low-density lipoprotein (LDL) cholesterol, triglycerides, total cholesterol, and Scr between the four GAR groups, but most patients that received alteplase before MT were in group2 or group3.

**Table 1:** Characteristics of AIS patients undergoing MT based on the GAR

	Group 1	Group 2	Group 3	Group 4	*P*
	(n = 136)	(n = 141)	(n = 139)	(n = 137)	
General data					
Age, Median, (IQR)	61 (52–71)	62 (54–71)	67 (60–77)	69 (61–79)	< 0.001
Male, n (%)	106 (77.9%)	100 (70.9%)	77 (55.4%)	85 (62.0%)	< 0.001
Smoking, n (%)	73 (53.7%)	57 (40.4%)	43 (30.9%)	35 (25.5%)	< 0.001
Medical history, n (%)					
Atrial fibrillation, n (%)	15 (11.0%)	18 (12.8%)	37 (26.6%)	31 (22.6%)	0.001
Previous TIA/stroke, n (%)	12 (8.82%)	27 (19.1%)	26 (18.7%)	46 (33.6%)	< 0.001
Cardiovascular disease, n (%)	11 (8.09%)	22 (15.6%)	19 (13.7%)	23 (16.8%)	0.158
Diabetes mellitus, n (%)	26 (19.1%)	32 (22.7%)	26 (18.7%)	52 (38.0%)	< 0.001
Hypertensive disease, n (%)	66 (48.5%)	63 (44.7%)	75 (54.0%)	90 (65.7%)	0.003
Clinical characteristics					
Systolic pressure, Median, (IQR)	130 (117–142)	134 (119–147)	136 (120–149)	140 (129–157)	0.001
Diastolic pressure, Mean ± SD	75 ± 13	77 ± 15	77 ± 17	79 ± 15	0.150
Median NIHSS score at admission (IQR)	12 (8–17)	15 (10–19)	16 (12–20)	17 (12–21)	< 0.001
Median NIHSS score at discharge (IQR)	6 (3–12)	7 (3–15)	6 (2–13)	8 (3–13)	0.550
Median Pre-stroke mRS (range)	0 (0–3)	0 (0–3)	0 (0–3)	0 (0–3)	0.168
Median Onset-to-reperfusion time, (min), (IQR)	481(277–741)	395 (303–629)	354 (274–520)	369 (268–585)	0.013
Stroke subtypes based on TOAST classification					0.362
Atherosclerosis n (%)	79 (58.1)	85 (60.3)	70 (50.4)	89 (65.0)	
Cardioembolism, n (%)	48 (35.3)	45 (31.9)	55 (39.6)	41 (29.9)	
Other determined etiology, n (%)	5 (3.7)	7 (3.7)	5 (3.6)	3 (2.2)	
Undetermined etiology, n (%)	4 (2.9)	4 (2.9)	9 (6.5)	4 (2.9)	
Baseline ASPECTS of anterior circulation strokes, Median, (IQR)	8 (6–10)	9 (7–10)	8 (6–10)	8 (7–10)	0.519
Baseline ASPECTS of posterior circulation strokes, Median, (IQR)	7 (4–9)	8 (6–10)	8 (6–9)	7 (5–10)	0.604
Laboratory data					
Fasting plasma -glucose, mmol/l	4.97 (4.42–5.46)	6.20 (5.75–6.82)	7.27 (6.82–8.01)	11.11 (8.98–14.42)	< 0.001
Glycated hemoglobin, %	5.8 (5.5–6.4)	6.0 (5.6–6.5)	5.8 (5.5–6.3)	6.2 (5.6–7.6)	0.007
Hemoglobin, g/l	135 (121–146)	132 (119–144)	131 (115–143)	131 (119–143)	0.308
GAR index	0.85 (0.76–0.90)	1.03 (0.98–1.08)	1.25 (1.18–1.32)	1.65 (1.50–1.94)	< 0.001
HDL cholesterol, mmol/l	0.95 (0.80–1.09)	0.98 (0.85–1.12)	0.98 (0.82–1.17)	1.03 (0.83–1.17)	0.199
LDL cholesterol, mmol/l	2.57 (1.99–3.23)	2.73 (2.16–3.34)	2.59 (1.98–3.13)	2.59 (1.91–3.34)	0.428
Triglycerides, mmol/l	1.23 (0.88–1.70)	1.20 (0.86–1.68)	1.19 (0.78–1.58)	1.11 (0.76–1.72)	0.418
Total cholesterol, mmol/l	4.47 ± 1.14	4.61 ± 1.14	4.36 ± 1.10	4.45 ± 1.22	0.339
Homocysteine, umol/l	9.10 (7.10–11.68)	8.30 (6.25–10.40)	8.50 (6.20–10.50)	7.65 (6.20–10.30)	0.005
Serum creatinine, umol/l	78.2 (63.8–90.9)	72.0 (61.0–89.0)	75.0 (62.0–91.2)	73.5 (62.0–91.3)	0.427
hsCRP, mg/l	3.78 (1.29–13.62)	3.80 (1.13–14.72)	5.60 (1.12–22.62)	9.72 (1.45–33.42)	0.016
INR	1.06 (1.01–1.12)	1.11 (1.03–1.19)	1.12 (1.05–1.24)	1.11 (1.03–1.20)	< 0.001
IVT, n (%)	25 (18.4%)	46 (32.6%)	46 (33.1%)	28 (20.4%)	0.004

*AIS* acute ischemic stroke, *MT* mechanical thrombectomy, *GAR* Glucose-to-glycated hemoglobin ratio; *Group 1* first Glucose-to-glycated hemoglobin ratio quartile, *Group 2* second Glucose-to-glycated hemoglobin ratio quartile, *Group 3* third Glucose-to-glycated hemoglobin ratio quartile, *Group 4* fourth Glucose-to-glycated hemoglobin ratio quartile, *IQR* interquartile range, *SD* standard deviation, *TIA* transient ischemic attack, *NIHSS* National Institutes of Health Stroke Scale, *mRS* modified Rankin Scale, *TOAST* Trial of ORG 10172 in Acute Stroke Treatment, *ASPECTS* Alberta Stroke Program Early CT Score, *HDL* high-density lipoprotein, *LDL* low-density lipoprotein, *hsCRP* high-sensitive C-reactive protein, *INR* international normalized ratio, *IVT* intravenous thrombolysis

### Association of GAR with mortality

**Fig. 2:**
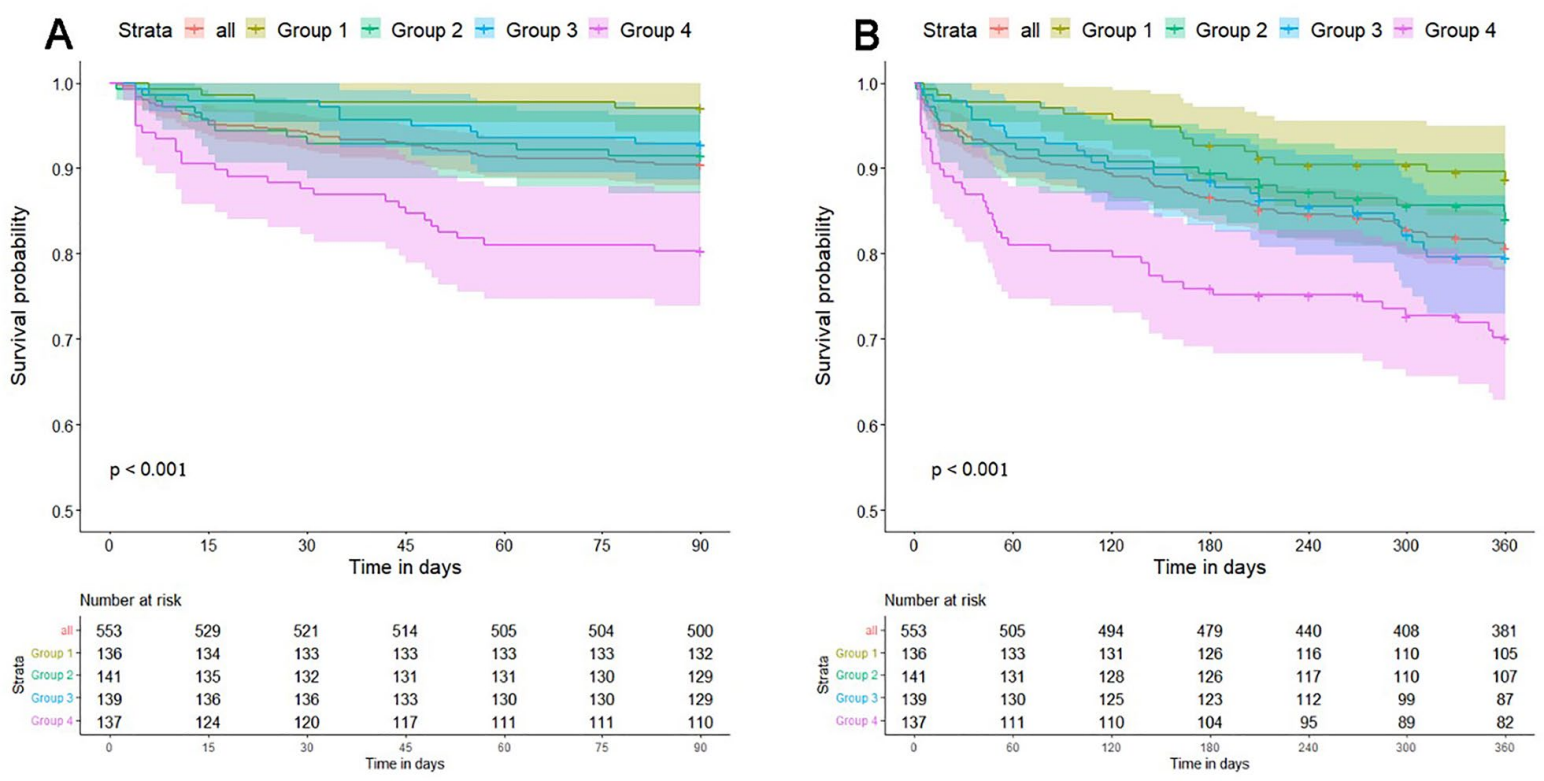
Kaplan–Meier survival curves in AIS patients after mechanical thrombectomy (MT). **A** Represented the survival probability during 3 months. **B** Represented the survival probability during 1 year

**Fig. 3:**
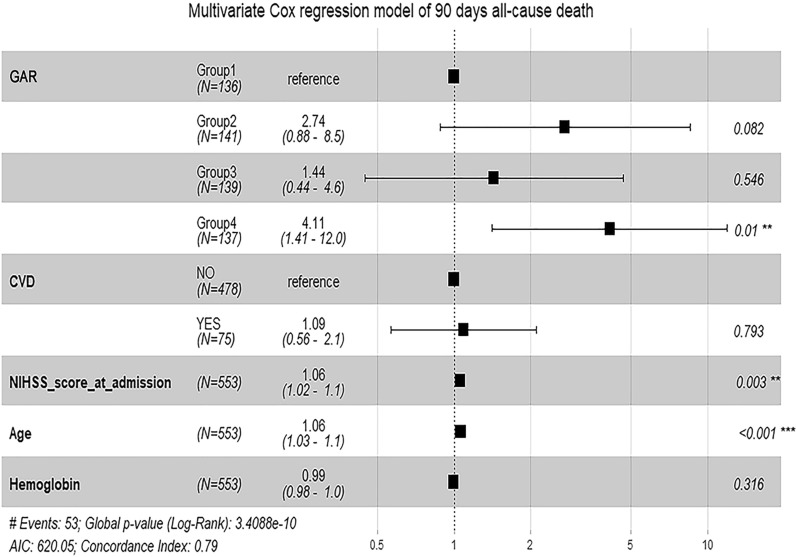
Multivariate Cox regression model of 90 days all-cause death. From left to right: variable name, group (number of people), HR (95% CI), and *P* value, respectively. *GAR* glucose-to-glycated hemoglobin ratio, *CVD* cardiovascular disease

**Fig. 4:**
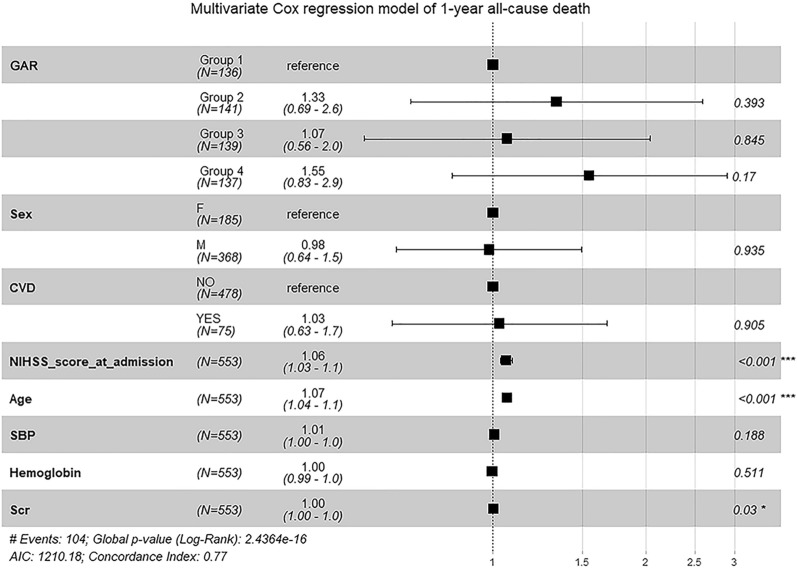
Multivariate cox regression model of 1 year all-cause death. From left to right: variable name, group (number of people), HR (95% CI), and *P* value, respectively. *GAR* glucose-to-glycated hemoglobin ratio, *CVD* cardiovascular disease, *NIHSS* National Institutes of Health Stroke Scale, *SBP* systolic blood pressure, *Scr* serum creatinine

The follow-up lasted for a median of 18 months (range 0–66 months). According to the Kaplan–Meier survival analysis, most of the deaths occurred within the first 3 months. 53 patients (9.58%) had died in 3 months, 102 patients (18.44%) had died in 1 year while 139 patients (25.13%) had died at the end of the follow-up (on December 31, 2021). Compared with group 1 of GAR, group 4 was associated with poor survival after MT in AIS patients (log-rank test: *P* < 0.001 for 3 month mortality and *P* < 0.001 for 1 year mortality) (Fig. 2). After controlling the potential confounders in Cox regression, we found that higher level of GAR was associated with an increased risk of 3 month all-cause mortality (group 4 vs group 1, HR 4.11, 95% [CI] 1.41–12.0, *P* = 0.01) (Fig. 3). However, no significant association was observed between GAR and risk of long-term all-cause mortality (group 4 vs group 1, HR 1.55, 95% [CI] 0.83–2.9, *P* = 0.17) (Fig. 4). And we could find that NIHSS score at admission (HR 1.06, 95% [CI] 1.03–1.1, *P* < 0.001), age (HR 1.07, 95% [CI] 1.04–1.1, *P* < 0.001) and Scr (HR 1.00, 95% [CI] 1.00–1.0, *P* = 0.03) are the independent risk factors for long-term all-cause mortality (Fig. 4). Therefore, Stress hyperglycemia may have a strong effect on the mortality of AIS patients with MT, especially on short-term all-cause mortality.

### Association of GAR with clinical outcomes

**Fig. 5:**
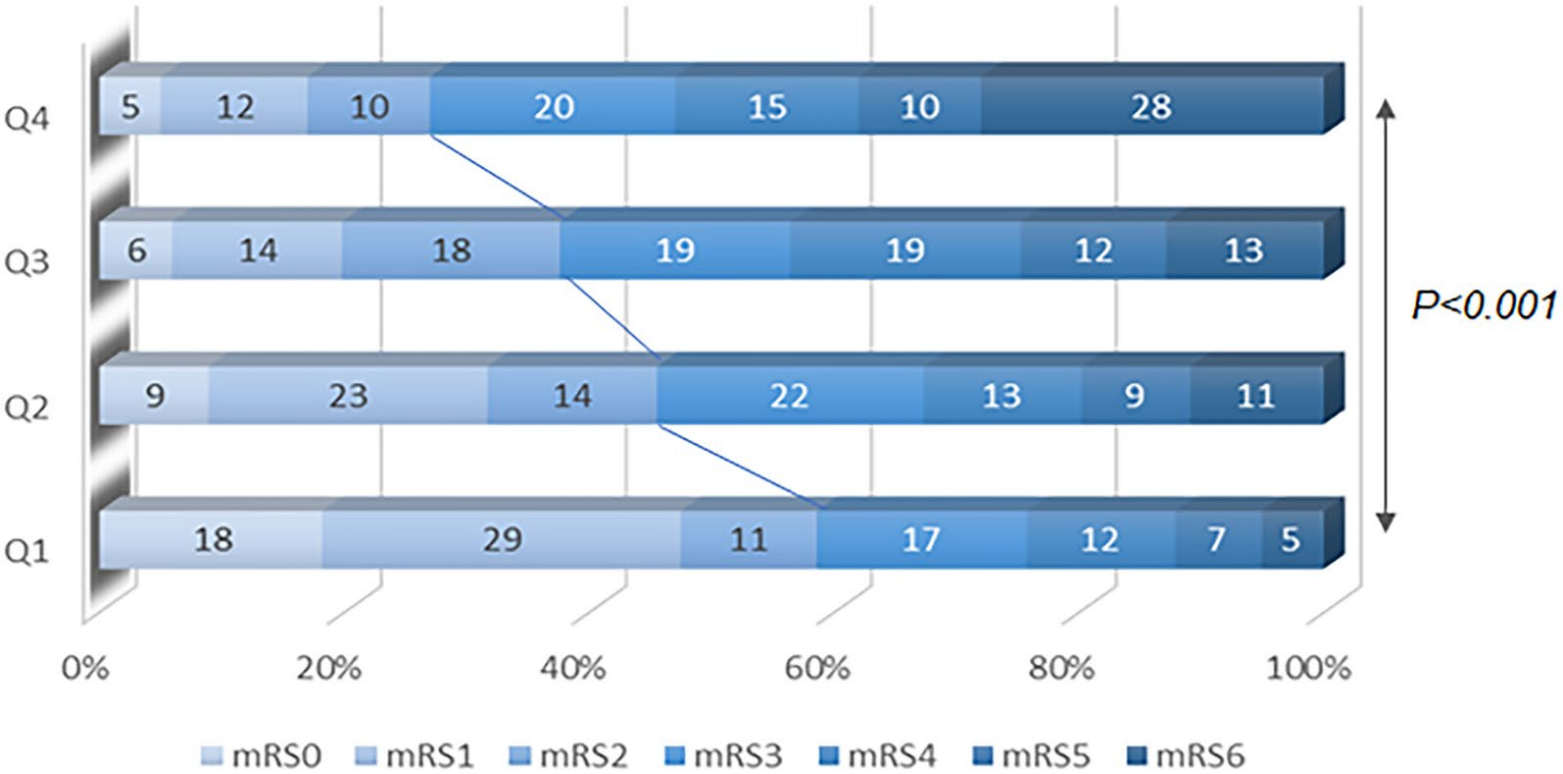
Functional outcome at 3 months. Modified Rankin Scale (mRS) scores for patients grouped by stress hyperglycemia ratio quartile

Table 2 lists the clinical outcomes during hospitalization and at 3 months of the patients with AIS after MT across GAR quartiles. The prevalence of END (12.5, 12.5, 21.9, and 53.1% for group1-4, respectively; *P* < 0.001 for trend), stroke-associated pneumonia (14.4, 21.6, 31.2, and 32.7%, respectively; *P* < 0.001 for trend), ICH (11.4, 29.5, 26.7, and 32.4%, respectively; *P* = 0.002 for trend) and 3 month poor functional outcome (17.6, 23.9, 27.4, and 31.1%, respectively; *P* < 0.001 for trend) differed significantly among the four quartiles. In addition, patients are more likely to gain adverse neurological outcomes as stress hyperglycemia increases (Fig. 5). However, the rate of getting sICH was not significantly different among the four quartiles.

**Table 2:** Association between GAR and Outcomes during hospitalization and at 3 months

Outcomes	GAR	Events, n (%)	Unadjusted OR (95% CI)	*P* value	Adjusted OR (95% CI)	*P* value
Early neurological deterioration†	Q1 (n = 136)	4 (12.5%)	Reference		Reference	
	Q2 (n = 141)	4 (12.5%)	0.49 [0.28;0.86]	0.96	1.08 [0.24;4.85]	0.916
	Q3 (n = 139)	7 (21.9%)	1.72 [0.49;6.95]	0.398	1.57 [0.45;7.01]	0.454
	Q4 (n = 137)	17 (53.1%)	4.52 [1.60;16.5]	0.003	4.47 [1.46;17.02]	0.014
	*P* for trend			< 0.001		0.005
Stroke-associated pneumonia‡	Q1 (n = 136)	30 (14.4%)	Reference		Reference	
	Q2 (n = 141)	45 (21.6%)	1.65 [0.97;2.85]	0.067	1.45 [0.84;2.54]	0.102
	Q3 (n = 139)	65 (31.2%)	3.08 [1.84;5.27]	< 0.001	2.22 [1.29;3.86]	0.002
	Q4 (n = 137)	68 (32.7%)	3.46 [2.06;5.92]	< 0.001	2.32 [1.34;4.06]	0.002
	*P* for trend			< 0.001		0.244
ICH#	Q1 (n = 136)	12 (11.4%)	Reference		Reference	
	Q2 (n = 141)	31 (29.5%)	2.88 [1.44;6.13]	0.002	2.39 [1.17;5.21]	0.020
	Q3 (n = 139)	28 (26.7%)	2.58 [1.27;5.53]	0.008	1.84 [0.89;4.02]	0.109
	Q4 (n = 137)	34 (32.4%)	3.37 [1.70;7.13]	< 0.001	2.63 [1.29;5.68]	0.010
	*P* for trend			0.002		0.108
sICH§	Q1 (n = 136)	3 (14.3%)	Reference		Reference	
	Q2 (n = 141)	9 (42.9%)	2.92 [0.83;14.1]	0.098	2.82 [0.80;13.06]	0.131
	Q3 (n = 139)	5 (23.8%)	1.62 [0.37;8.54]	0.522	1.59 [0.37;8.08]	0.541
	Q4 (n = 137)	4 (19.0%)	1.32 [0.27;7.26]	0.731	1.44 [0.30;7.60]	0.647
	*P* for trend			0.917		0.532
Three-month poor functional outcome (mRS 3–6) ‖	Q1 (n = 136)	56 (17.6%)	Reference		Reference	
	Q2 (n = 141)	76 (23.9%)	1.67 [1.04;2.69]	0.035	1.47 [0.86;2.53]	0.155
	Q3 (n = 139)	87 (27.4%)	2.38 [1.47;3.89]	< 0.001	1.45 [0.83;2.54]	0.190
	Q4 (n = 137)	99 (31.1%)	3.70 [2.24;6.19]	< 0.001	2.06 [1.13;3.77]	0.018
	P for trend			< 0.001		0.013

*CI* indicates confidence interval, *GAR* Glucose-to-glycated hemoglobin ratio, *OR* odds ratio, *ICH* intracranial hemorrhage, *sICH* symptomatic intracranial hemorrhage, *mRS* modified Rankin Scale

^†^Adjusted for age, sex, and NIHSS score at admission

^‡^Adjusted for age, NIHSS score at admission, previous TIA/stroke, smoking, and hsCRP

#Adjusted for atrial fibrillation, NIHSS score at admission, homocysteine, and IV tPA administration

^§^Adjusted for NIHSS score at admission, and IV tPA administration

‖Adjusted for age, sex, systolic pressure, NIHSS score at admission, pre-stroke mRS, cardiovascular disease, previous TIA/stroke, smoking, hemoglobin, and serum creatinine

After controlling for potential confounding factors, there remained a clear relationship between high GAR and END (group 4 vs group 1, OR 4.47, 95% CI 1.46–17.02, *P* = 0.014), stroke-associated pneumonia (group 4 vs group 1, OR 2.32, 95% CI 1.34–4.06, *P* = 0.002), ICH (group 4 vs group 1, OR 2.63, 95% CI 1.29–5.68,* P* = 0.010) and 3 month poor functional outcome (group 4 vs group 1, OR 2.06, 95% CI 1.13–3.77, *P* = 0.018). Furthermore, patients with presenting hyperglycemia exhibited a tendency toward END within 24 h (*P* for trend = 0.005) and poor functional outcome at 3 months (*P* for trend = 0.013). We did not detect any difference between GAR and sICH (group 4 vs group 1, OR 1.44, 95% CI 0.30–7.60,* P* = 0.647).

### AUCs of GAR for predicting 3-month mortality and 1-year mortality

**Fig. 6:**
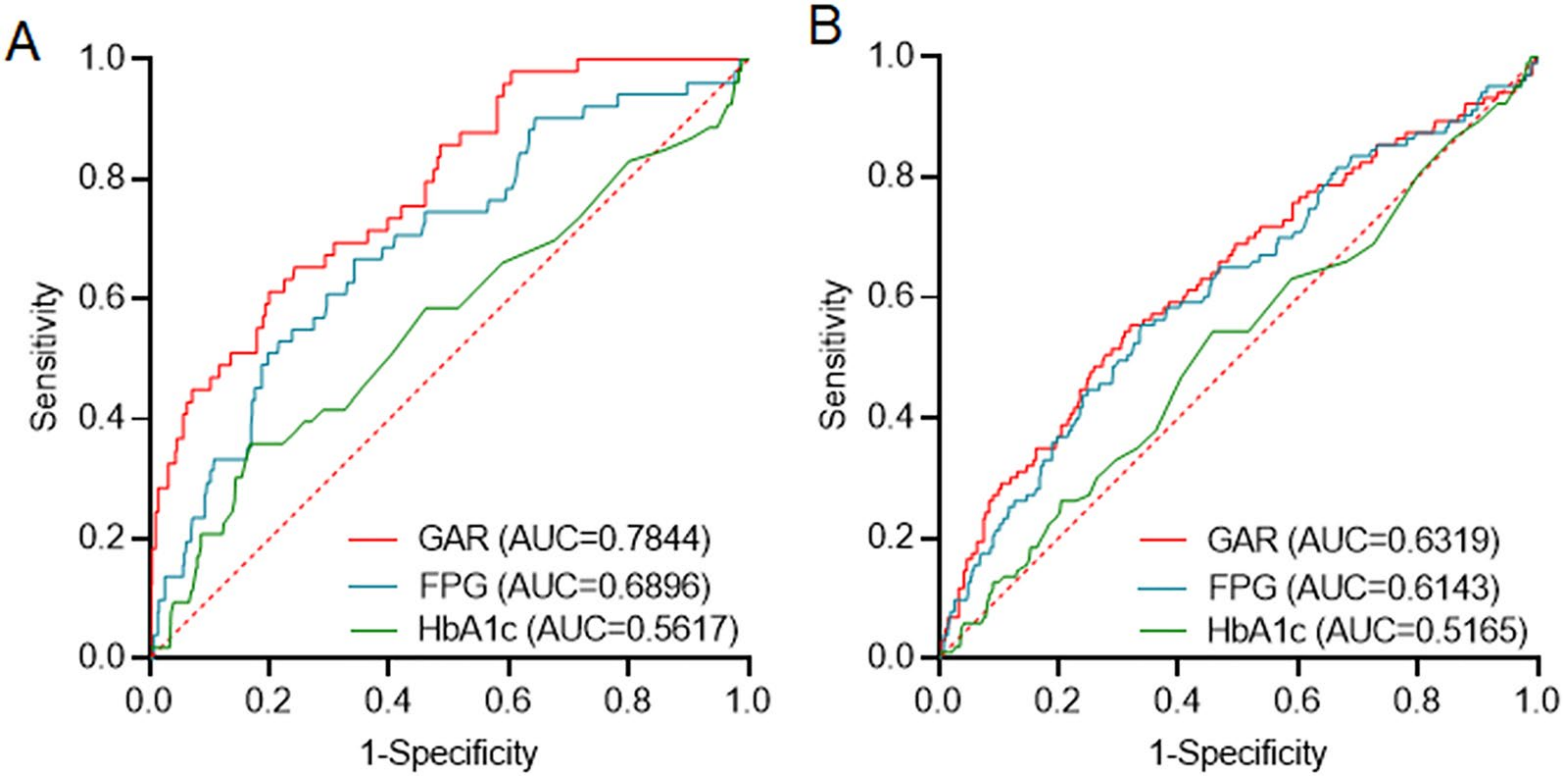
Receiver operating characteristic curve (ROC) analyses of the glucose-to-glycated hemoglobin ratio (GAR), admission fasting plasma glucose (FPG), and HbA1c for predicting 3-month mortality (**A**) and 1-year mortality (**B**)

The AUC of GAR for predicting 3 month mortality after MT in AIS was 0.7844 (95% CI 0.7199–0.8489, *P* < 0.0001); the AUC for FPG was 0.6896 (95% CI 0.6117–0.7674, *P* < 0.0001), and that for HbA1c was 0.5617 (95% CI 0.4720–0.6513, *P* = 0.1396). The AUC of GAR for predicting 1 year mortality after MT in AIS was 0.6319 (95% CI 0.5690–0.6948, *P* < 0.0001); the AUC for FPG was 0.6143 (95% CI 0.5518–0.6768, *P* = 0.0003), and that for HbA1c was 0.5165 (95% CI 0.4531–0.5798, *P* = 0.6022) (Fig. 6). The AUC analysis showed that GAR had a better predictive value for 3 month and 1 year mortality than did FPG or HbA1c.

## Discussion

This study analyzed data from 553 consecutive patients with AIS who underwent MT. The results showed that GAR was significantly associated with 3 month mortality after MT in patients with AIS, and hence that GAR may be a useful predictor of short-term mortality after MT in AIS. Elevated stress glucose, as quantified by a higher GAR, was found to be associated with increased risks of END, stroke-associated pneumonia, ICH and 3 month poor functional outcomes. Stress hyperglycemia appears to play a major role in short-term outcomes, but was not significant associated with long-term mortality.

A number of previous studies have found that hyperglycemia is a predictor of hemorrhagic transformation and poor outcome in AIS patients and is associated with increased disability and mortality [23–25]. Hyperglycemia in AIS patients may be associated with worse outcomes and increased in-hospital mortality with diabetes mellitus [26]. At present, there is no uniform definition of stress hyperglycemia. GAR can reflect the true elevation of blood glucose levels in acute disease by correcting the basal blood glucose level. Such relative measures that also consider background glucose are better predictors of prognosis in critical illness compared with using absolute hyperglycemia [9, 11].

Stress hyperglycemia, as measured by the glucose-to-glycated hemoglobin ratio, is associated with an increased risk of stroke recurrence and all-cause death in AIS patients [12]. Our previous studies have shown that stress hyperglycemia ratio, as measured by FPG (mmol/L) divided by HbA1c(%), was associated with a reduced rate of excellent neurological outcomes in patients with anterior circulation stroke after MT regardless of diabetes status [27]. Several studies have shown a significant association between admitted hyperglycemia and adverse short-term outcomes of AIS patients after MT [23, 28]. Elevated glucose-to-glycated hemoglobin ratio as a stress hyperglycemia in AIS patients undergoing MT is associated with a 3 month risk of adverse outcomes and death [10, 29]. However, the study did not compare GAR with long-term mortality. Zhu et al. found that stress hyperglycemia, as measured by the FPG (mmol/L)/HbA1c (%) ratio, was associated with an increased risk of all-cause mortality at 3 and 12 months after AIS patients [12]. However, the study used logistics regression analysis and included AIS patients but no MT. Schmitz et al. have shown a significant effect of stress hyperglycemia on short-term mortality in patients with myocardial infarction, but no effect on long-term mortality [30]. A plausible explanation might be that, by definition, stress hyperglycemia is a transient dynamic disorder that responds to changes in acute events, such as acute disease progression. Conversely, severe comorbidities associated with hypertension or diabetes may play a more important role in long-term outcomes.

Postoperative hyperglycemia is associated with an increased risk of postoperative bleeding complications in AIS patients with MT [31]. The present findings further confirm these data, showing that stress hyperglycemia in AIS patients treated with MT as measured by GAR is related to the occurrence of ICH. The mechanisms underlying the associations of GAR with stroke severity and prognosis in patients with AIS remain unclear. Several mechanisms that have been proposed may account for the association between stress hyperglycemia and poor outcomes in patients with AIS treated with MT. Hyperglycemia causes vasoconstriction by damaging vascular endothelial cells, inhibiting nitric oxide production, and stimulating the release of thromboxane A2, thereby causing an imbalance between vasoactive and prothrombotic substances, and resulting in reduced blood flow [32]. Patients with AIS in the presence of hyperglycemia have reduced plasma fibrinolytic activity and increased levels of inhibitors of fibrinogen activator, which may reduce vascular recanalization [33]. Hyperglycemia may exacerbate brain injury by promoting oxidative stress and inflammatory responses, inducing reactive oxygen species and free-radical generation, promoting ischemic cell death related to hemidesmosomes, and aggravating brain edema [34–36]. Hyperglycemia in AIS patients treated with MT aggravates oxidative stress and endothelial cell damage, causing reperfusion injury to brain tissue, thus leading to a poor patient prognosis [37, 38]. Giovanni Merlino et al. reported that stress hyperglycemia is a modifiable predictor of futile recanalization in patients with large vessel occlusion treated with MT [39]. Hyperglycemia may promote futile recanalization by through lactate production and intracellular acidosis, disrupting the blood–brain barrier and affecting cerebrovascular autoregulation to cause reperfusion injury and hemorrhagic transformation [40, 41]. Therefore, it increases the risk of futile recanalization in AIS patients with large vessel occlusion after MT therapy.

Admission hyperglycemia (> 7.8 mmol/L) was found to be associated with poststroke infection [42]. The present study shows that compared with group 1 of the GAR in patients with AIS who received MT, a higher GAR is associated with a higher risk of stroke-associated pneumonia. It has been found that stroke-induced immunosuppression exists in AIS patient [43]. The increased activity of the hypothalamic–pituitary–adrenal axis after stroke was accompanied by an increase in the cortisol concentration, which stimulated the secretion of anti-inflammatory cytokines [44–46]. The current studies indicates that stroke-induced immunodeficiency is achieved by activating the sympathetic nervous system and the HPA axis to inhibit the body’s cellular immune function after stroke, thereby enhancing the susceptibility to infection [47]. There is controversy about the causal relationship between stress hyperglycemia and stroke-associated pneumonia caused by stroke-induced immunodeficiency. Stress hyperglycemia may trigger a vicious cycle that further exacerbates the development of stroke-related pneumonia, and so further research is needed.

However, there are two limitations of this study that should be noted. First, this study had a single-center retrospective cohort design, and so selection bias may have reduced its predictive value. Second, the relatively small sample reduced the statistical power. Future studies involving multiple centers and larger samples need to be performed.

## Conclusions

Stress hyperglycemia, quantified by a higher GAR, is associated with all-cause mortality and poor functional outcomes in patients with AIS who undergo MT. Furthermore, GAR may contribute to improving the predictive efficiency of all-cause mortality in patients with AIS after MT, especially short-term all-cause mortality.

## Data Availability

Not applicable.

## Abbreviations

GAR: Glucose-to-glycated hemoglobin ratio
AIS: Acute ischemic stroke
MT: Mechanical thrombectomy
FPG: Fasting plasma glucose
HbA1c: Hemoglobin A1c
HR: Hazard ratio
CI: Confidence interval
mTICI: Modified thrombolysis in cerebral infarction
CT: Computed tomography
MRI: Magnetic resonance imaging
IVT: Intravenous thrombolysis
mRS: Modified Rankin Scale
ORT: Onset-to-recanalization time
NIHSS: National Institutes of Health Stroke Scale
END: Early neurological deterioration
ICH: Intracranial hemorrhage
sICH: Symptomatic intracranial hemorrhage
ECASS II: Second European–Australasian Acute Stroke Study
KM: Kaplan–Meier
CVD: Cardiovascular disease
SBP: Systolic blood pressure
Scr: Serum creatinine
AUC: Area under the receiver operating characteristic curve
HRs: Hazard ratios
ORs: Odd ratios
TOAST: Trial of ORG 10172 in Acute Stroke Treatment
ASPECT: Alberta Stroke Program Early CT Score
HDL: High-density lipoprotein
LDL: Low-density lipoprotein
